# Maintaining respect and fairness in the usage of stored shared specimens

**DOI:** 10.1186/1472-6939-14-S1-S7

**Published:** 2013-12-19

**Authors:** Takafira Mduluza, Nicholas Midzi, Donold Duruza, Paul Ndebele

**Affiliations:** 1 University of Zimbabwe, P.O. Box MP 167, Mount Pleasant, Harare, Zimbabwe; 2 Research Council of Zimbabwe, P.O. Box CY 294, Causeway, Harare, Zimbabwe; 3 Zimbabwe Revenue Authority, PO Box CY 803, Causeway, Harare, Zimbabwe; 4 Medical Research Council of Zimbabwe, P.O. Box CY 573, Causeway, Harare, Zimbabwe; 5 College of Health Sciences, University of KwaZulu-Natal, Durban, South Africa

**Keywords:** biological samples, sharing specimens, research collaboration, ethics, regulation of research, resource limited

## Abstract

**Background:**

Every year, research specimens are shipped from one institution to another as well as across national boundaries. A significant proportion of specimens move from poor to rich countries. Concerns are always raised on the future usage of the stored specimens shipped to research insitutions from developing countries. Creating awareness of the processes is required in all sectors involved in biomedical research. To maintain fairness and respect in sharing biomedical specimens and reserch products requires safeguarding by Ethics Review Committees in both provider and recepient institutions. Training in basic ethical principles in research is required to all sectors involved in biomedical research so as to level up the research playing field.

**Discussion:**

By agreeing to provide specimens, individuals and communities from whom samples are collected would have placed their trust and all ensuing up-keep of the specimens to the researchers. In most collaborative set-up, laid down material transfer agreements are negotiated and signed before the shipment of specimens. Researchers, research ethics committees (RECs) and institutions in the countries of origin are supposed to serve as overseers of the specimens. There is need to advocate for honesty in sample handling and sharing, and also need to oversee any written commitments by researchers, RECs and institutions at source as well as in recipient institution. Commitments from source RECs and Institutional Review Boards (IRBs) and in the receiving institution on overseeing the future usage of stored specimens are required; including the ultimate confirmation abiding by the agreement. Training in ethical issues pertaining to sample handling and biomedical research in general is essential at all levels of academic pursuit. While sharing of biological specimens and research data demands honesty and oversight by ethical regulatory agents from both institutions in developing country and recepient institutions in developed countries.

**Concluding summary:**

Archiving of biological specimens requires reconsideration for the future of biomedical findings and scientific break-throughs. Biomedical ethical regulations still need to established clear viable regulations that have vision for the future of science through shared and archived samples. This discussion covers and proposes essential points that need to be considered in view of future generations and scientific break-throughs. The discussion is based on the experience of working in resource-limited settings, the local regulatory laws and the need to refine research regulations governing sharing and storage of specimens for the future of science.

## Background

Conditions and the proper working of International Ethics Committees (IECs), National Ethics Committees (NECs) and similar bodies may differ in size, composition, working methods and mandates, but the impact of the ethics committees on public debates and legislation depend on the knowledge, wisdom, commitment and willingness to work of the members. The goals of national & international ethics committees include identifying important ethical issues, facilitating the ethical debates in their countries by providing relevant background material and advancing sound reasons of recommendations on what to do and what to avoid in difficulty and complex issues. NECs base their conclusions on a broad value judgment combined with relevant information not only on just scientific information, but also on people's attitudes, existing regulations, societal trends, economic conditions and other consequences [1-4]. Since bioethics is not a subject that is taught in many universities around the world, there is need to promote, disseminate and elaborate on the bioethical principles laid down in all regulatory documents like Council for International Organization of Medical Sciences, Belmont report, Nuffield Council on Bioethics, World Medical Associations, etc [1-5]. Research is carried out in a scarce-resource population, mainly through public institutions and links with researchers. This is a very important issue because it has an impact on informed consent as well as on benefit sharing and the social relevance of the research conducted [5].

Research on genetics and the storage of body tissue (biobanks) have increased the work and challenges faced by REC or IRBs. NECs regulate the functioning of RECs in the country and to develop or strengthen the legal framework governing research with human participants [5,6]. Generally, most resource-constrained areas/communities have banned the export of biological materials until a legal framework is established [7]. While this step is important for the health authorities in several countries, this poses in turn great challenges to those whose research requires the export of samples to other countries. The researcher fraternity is somehow disturbed with reports indicating that some IRBs are rejecting research where the storing of coded samples form an integral part of the study [8]. Such protocols were rejected because the IRBs were concerned that the participant protection was not guaranteed and also due to likely uncontrolled use of the biological materials at the recipient institution. The concerns were justified as the participants that provided the samples may have not fully understood the range of tests that can be done on their samples. While researchers are expected to behave in ethical manner, it cannot be ruled out of the extent the researchers can go with all the samples at their disposal [7]. Based on the type and magnitude of some research being approved and conducted in poor and developing communities, it may be highly possible that some researchers conduct seemingly minor or simple harmless studies just to get access to some samples for storage, export or use in unethical or unapproved research. Speculation cannot be avoided in which some researchers sell samples to laboratories unknown to IRBs/RECs. Given the magnitude of the information that scientists can derive from stored specimens. As such, RECs/IRBs need to be concerned and wary when issuing out approvals, especially to a collaborative group with institutions scattered all over the world.

Most developing countries would need to conduct high-tech scientific research but due to poverty and unavailability of some basic equipment, limitation exists. While institutions in developed countries that show intent to do research in developing countries offer a good solution, challenges still exits on the modalities. This is so often because they meet the cost of the study and provide expertise. However, specimens are usually shipped out of developing country under the pretext of lacking facilities for storage and equipment to run advanced analysis. Researchers in developed countries benefit far much more from most types of collaboration that they are often unwilling to invest in strengthening capacity of the weaker institution in the host country [6,9]. A trend has appeared in the research arena almost like modern day of colonization through sharing regions or areas of resource poor communities. Moreover, some research proposal and protocols are submitted to IRBs/RECs without local investigators until advised and pointed out by the reviewing teams [9,10]. Very often it is likely that due to pressure on academic institutions to conduct research and publish their findings as a requirement for promotion, has tempted some researchers to compromise on ethical standards, resulting in great challenges posed by biobanking and the export of samples. However, regulations should be made flexible to accommodate rapid scientific advances [8,11]. Sometimes stringent measures may be required to protect these samples, but such measures may not be good for the future of science, hence paving way for the need to balance during protocol review process [12,13], and taking cognizance of the need to be able to track the evolution of the diverse disease conditions for the sake of the future discoveries or unraveling of current disease challenges [8,13].

Biomedical research has led to significant improvement and advancement in medical field and more is still needed to further improve preventive, diagnostic and therapeutic approach including proper ways to tackle emerging health related challenges [11,14-16]. In this regard, the research participant need to be protected as well as setting acceptable regulations in the research arena involving researchers, coworkers and collaboration, especially putting in place more emphasis on participant and the researcher in resource-limited areas. There are several ethical and practical challenges faced by researchers that normally go unresolved, especially affecting researchers and participants in developing countries. The discussion involves ethical and practical challenges encountered in resource-constrained settings, and explores some possible ways to overcome the challenges. Further, giving analyses of the challenges surrounding sharing products of the research collaboration. Hoping that the discussed issues will go a long way sensitizing investigators and the related stakeholders on the need to operate within the research ethical framework for both the participants and the researchers and further assist in setting up relevant ethical guidelines or legal frameworks.

Currently, Africa is faced with several diseases that are playing havoc on socioeconomic development, giving an environment highly fertile and conducive for biomedical research on its inhabitants [11,15,16], and also likely opportunity for collaboration that can exploit the available experts, researchers and their resources. Africa still has massive deficit in health research capacity in numbers and competencies, underdeveloped research institutions compounded by poor networking opportunities for collaboration. Collaborative research has been on the increase; there are now an increased number of institutions that enable research scientists from developed countries to undertake research [9,10,14,17]. In turn there has been an upsurge of institutional and governmental agents that purportedly support research but have come up with stringent regulations to monitor flow of data and samples between collaboration. In certain countries the governmental department of customs and exercise has been included in monitoring the flow of this essential research commodity. All this indicating that the research landscape across Africa has changed considerably where there are now locally owned and led research institutions [9]. These have institutional research ethics committees that require standardization in as far as operation is concerned in sample sharing during collaboration.

At times non-governmental players based in both developed and developing countries that are involved in community based activities have assisted in advocacy, mobilizing funds to enable training in an attempt towards increased attention to good collaboration and sharing the products of research [9]. Some are engaged in capacity strengthening of health research institutions and researchers in developing countries. Strengthening of research capacity and the inclusion of training in universities and colleges curricula may go a long way in empowering the limited resources research for decision making and demanding equal collaboration and sharing of research products. Although there has been considerable advancement in resolving the 10/90 gap, particularly with respect to communicable diseases, the gap maybe widening due to the rapid technological advancement in developed countries and the ever increasing epidemiological diseases of infectious and non communicable nature [11].

Some institutions in resource-limited areas have strong leadership and almost a universal collaboration policy, even though this appears as the aftermath of the scramble for study sites in developing countries that resulted in research institutions and some universities located in developed countries sharing the research sites situated in resource-limited areas. A considerable investment has been achieved in setting up IRBs and NEC for regulation purposes and to protect study participants [18]. Much efforts should be made to link various researchers and research endeavours by establishing collaborative multidisciplinary teams, links and consortia that should be able to address research problem holistically [9]. There is also the need for increased numbers of researchers and research institutions capable of creating research agenda and operational guidelines on sharing specimens and research products, increased collaboration and adapting research findings into policy [14]. Qualified researchers and managers should be able to mentor and train juniors, develop and sustain a fruitful research culture, manage risk, be accountable and trustworthy. Establishing strong collaboration with northern partners and sharing unwinding of good collaboration policies with their southern partners in an effort to make uniform policies. Most NECs, IRBs and ERCs recently established over the last two decades have already contributed immensely to ongoing clinical and field trials and also to the future ones and even to the improvement of health research systems, while more should be invested in sharing the products of research.

Such set-ups have made the research fraternity realize the benefits of research regulations, bringing in sanity to mostly a deceptive environment in collaboration. While in the process of contributing to resolving health challenges, the research jungle seems to ultimately get tamed. However, not much has been realized along specimen sharing between resource-limited areas and highly financed institutions in developed countries. The challenges ushers in the need to address at an early stage on how southern research partners and their communities would derive benefits from such research and participation. Also taking into consideration the future of science from the viewpoint on the need for biobanks. There is also need for proper review of the research protocol and ethical issues, the need for training researchers and associated personnel, mentorship, develop institutional leadership personnel with a wide vision and able to play a major role as members of committees to review study designs and provide clinical and field project oversight. In particular, where some of the neglected diseases that need health research attention are endemic, research is conducted only in remote areas, which are difficulty to reach and rarely are closely monitored by the in-country regulatory authorities due to resource limitation for making follow-up.

### The ethics on research collaboration

Several research programmes have exhibited traces of deception to the vulnerable collaborative researchers or/and participant in resource-limited areas. Exploitation of the vulnerable researcher or participant is normally manifested in coercive or deceptive research implementation strategies. Most of these exploitative activities go unnoticed where research oversight is not well established. The remedy for exploitation in research lies in thoroughly and adequately well-informed research participant [18,19], combined with procedures that respect the cardinal principles of autonomy, beneficence, non-maleficence and justice [1-4]. A high burden of disease, combined with desperate poverty and ignorance make people in resource-limited settings highly vulnerable to exploitation [14]. Populations of resource-constrained settings are highly vulnerable in biomedical research, while research in such settings in justifiable to solve the urgent health challenges, care must be taken to avoid harming, exploiting or otherwise treating research participants in unethically acceptable ways [7,9,17].

Shared specimens play a very important role in the future of sciences. Imagine the discovery of HIV from the archived samples that later provided all the clues after the patients who provided the samples and clinical history had long deceased. The archived samples contributed to unraveling the mysterious infection that had devastated certain sectors of the world population to be discovered [20-22]. Places all over the world have unique health challenges that call for biobanking, necessary for continuing investigation. The ethical issues surrounding such commodity in research may demand sculpturing operational codes that should bring in rational sharing of such important research study materials. However, due to limited resources in developing areas that lack capacity to plan ahead and fail to establish storage facilities, a greater number of specimens are shipped to developed countries with well established and resourced research facilities and experts [6,12]. However, are these specimen ethically and legally collected with the full knowledge in the collaborative research and by the regulatory authorities?

**Figure 1:**
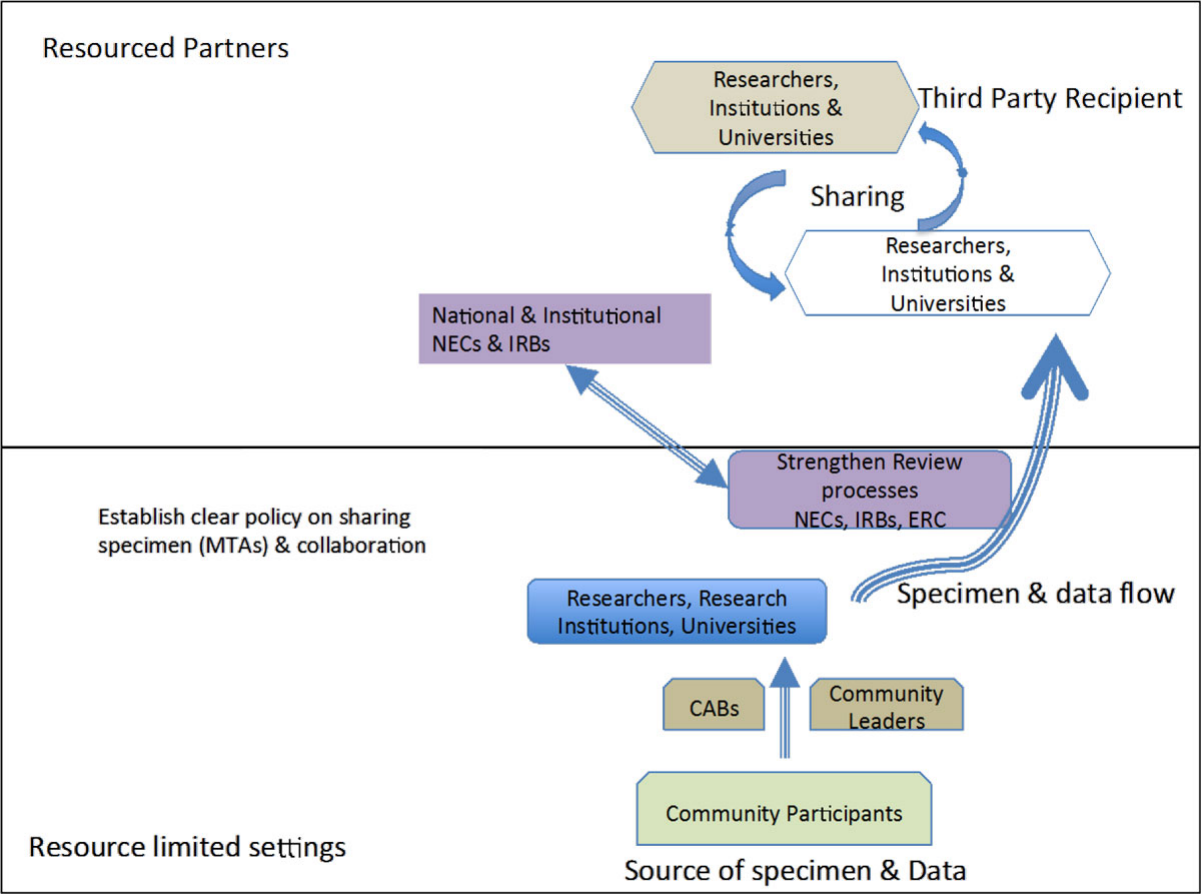
**Showing the flow of data and specimen from resource limited settings to resourced and developed centres**. The top part showing resourced centres may even create further specimen exchange that does not involve the researchers and the participating institution from developing countries. National ethics committees in resource limited areas need to establish links with corresponding national and institutional ethics committees in countries where samples are shipped.

Most of the genetic epidemiological studies may be based on data of the diseases or traits derived from families, ethnic groups, communities or populations. To understand such trends and traits involve use of blood samples for DNA extraction that may generate databases with genetic phenotypic, clinical and socio demographic data [13]. Although participants may give informed consent in their own capacity, their involvement in such studies could affect communities, ethnic groups or the populations in diverse ways. In the case of international collaborative studies that involve sharing of specimen or simple shipping samples, usually from South to North or to high technology laboratories. Some materials may either be collected prospectively or the materials extracted from stored samples. The use of stored human samples is a challenge that has sparked debate globally [6,20]. The stored samples could have accumulated from routine diagnostic and treatment activities of health institutions or from heath research conducted over several years back. Due to the ethical, legal and socio-economic issues surrounding such product of research activities in resource limited settings, there is need to come up with progressive legislative and regulatory framework aimed at addressing such issues. Currently, most international collaborative research projects focusing on diseases of the poor collect specimens from developing countries and calls for the development of necessary framework for research using archived samples. A simple interrelationship is shown in Figure 1, portraying stages that can be strengthened to rationalize ethically sharing of specimens. However, some developing countries have now come up with repressive regulations that will do injustice to the future of science.

Although some tests may not be available or affordable to the majority of populations in developing countries, there is no justification in using such pretext to shipping biological materials to highly developed countries for the sake of future access to these valuable materials. Some research institutions in developed countries have been shipping such valuable materials over a very long period without revisiting to solve the source of the identified weaknesses, thereby showing insincerity in their activities and excuses. Sometimes samples may be collected prospectively, ideally after obtaining ethical approval from relevant Ethics Review Committees and informed consent from individuals. It is normally important for researchers to ensure that the informed consent captures all issues that are concerned to the participants and their communities [6,12,18]. All such concerns need to be addressed upfront with the target community, in advance of sample targeted for shipping. Note that prospective collection of samples for archiving requires dealing with pertinent ethical and practical issues upfront and extreme openness. Some archived samples from health institutions may be appropriate but again there are some legal ethical issues to be addressed early. Archived samples from previous research projects are a common feature of teaching hospitals where some clinicians undertake research as well as treatment management. There are also samples stored in national and private commercial biobanks, even though rare in resource constrained areas. Regardless of the source of samples, general personal identifiers may be delinked such as participant names, address, birth, hospital admission number or national registration number but still the samples may be linked with community, village, or country [19], hence clear ethical approval is necessary.

#### Ethical issues surrounding sharing of samples

Researchers have the obligation to design protocols that discloses all the critical information about the study, giving the prospective participant to understand the potential risks, potential benefits [19]. While voluntariness in the informed consent may be compromised in as far as archiving of some samples from resource limited settings, it is difficulty to justify use of samples collected long back for a current investigation [8,13]. This has impact on the progress of science, making use of any available material to understand the challenges that face human health in future. Consent for stored samples for future research really circumvents such obstacles in which the use of such samples is not diseases specific but is essentially important to unravel the problem from health challenges. Sometimes researchers ask for consent to use collected samples in yet to be designed experiments. Giving an open-ended consent is analogous to giving a blank signed cheque to researchers [20]. There is need to sensitize Ethics Review Committees in developing countries to deal with the informed consent for future specimen use. Commonly, scarcity of information exists in developing countries on the best practice to address such issues. Sharing of specimens and future use require in-depth consideration and analysis by the responsible authorities during review of protocols, consent forms as well as relevant regulatory frameworks that play a part in the approval of proposals. Due to the nature of certain research studies, community engagement may help to ensure that there is awareness about the research at community level and not just at individual level. The community from which the individual sample is collected should know the intended archiving for non-specific investigation in future [13,18,20]. Normally, the ordinary community participant is sidelined while the research ethics issue is addressed along some research activities for academic purposes.

The shared or archived specimens have the potential to generate new knowledge that may not have direct benefits to the time of the sample collection. Whereas, some research activities such as clinical trials may benefit the study participants directly, archived specimen tends to generate knowledge, which may not lead to some intervention immediately. In addition, although the research outcome of the future endeavor using stored specimens could generate generalizable knowledge that could benefit mankind, but not direct benefits are realized by the researchers involved in the initial work where samples were collected [20]. The benefits are generally realized by the stronger group (Figure 1) in the developed country where the archiving is done and the driving sponsor of such endeavor who in most cases may be pharmaceutical company with commercial benefits, while the donor may not even access the commercial products derived from research testing on their samples [14].

#### Collaboration and sharing of research products

Another contention in sharing of samples and data between researchers and their institutions is openness during future use of stored specimens or data. Epidemiological studies focusing on diseases of the poor countries bring together collaboration between researchers from North in collaboration with those in South; or sometimes some consortia or many institutions are formed so as to achieve large sample sizes quickly within reasonable time. The collaborators from developed countries usually are the leaders of the research grants and may possess the technology, and end up in full control of all the collected data at the end of a given study. The benefits are only discernible during the active study while later, the researchers from resource-limited areas are not included as part of the studies on the archived specimens (Figure 1). Such occurrences reveal a lack of capacity in most developing countries to store large quantities of samples on a long-term basis and for future use. Most developing countries do not have the capacity to store large quantities of samples on a long-term basis and for future investigative activities hence samples are shipped to collaborative centres in developing countries for storage.

The developing countries or resource-limited areas play the role of sample collection ground, even though the benefits derived from research may eventually spread to all parts of the world, only would continuous benefits be realized if such areas were capable of sample storage and analysis. The challenges require immediate addressing right from drawing up the protocols on sample sharing and the research outcome of the data (Figure 1). The informed consent should explicitly explain all pertinent issues early in the study, should explain the rationale behind the need to store samples for future use. Another aspect of minimizing conflict and deception is to have well-trained ethics review committees capable of critically reviewing the pertinent issues during review process. After approval, the communities need to be empowered to be able to monitor the research with the monitoring extending beyond the life span of the approved project. The ERCs should be vigilant while analyzing protocols with indication for future research that may inappropriately be based on stored samples and ethical approval given for previous investigations. Resource constrained areas need to empower national regulatory authorities and set up regulation that are particularly relevant to the developing world perspective and may not address some of the issues prevailing in the settings. Very often there is need to establish material transfer agreement that may regulate and make it known of the intention for archived samples.

Every research protocol that would involve sharing samples require establishing of these regulations upfront, stating all details as the number of the samples to be transferred, the purpose for which the samples should be used, the research institution permitted to use the samples, whether or not they may be stored for possible future research and the details of such future research. The clarity should include whether the personal identifiers should be removed before transfer. Communities need to be engaged right from start and all details revealed of the intention. There are various models of community engagement and researchers have to find out models that would be acceptable in the particular communities where the study is to be conducted. This has to be emphasized that community engagement should be well planned upfront, included in the budget and in the project timeframe just like other project activities.

Further, members making up regulatory authorities require continuous training to understand the benefits in shared samples. Regulations of studies and protocols require to appreciate the knowledge that can be generated from stored specimen including pre-symptomatic detection of some predisposition condition to certain diseases that may required archiving of specimen where such facilities exists for future testing. The regulatory authorities probably would need to incorporate technology transfer and capacity building to go along with transfer of samples and sharing of specimens. Wherever possible, efforts should be made to transfer relevant modern technology from developed countries to resource constrained areas to enable full utilization of all research materials without resort to shipping samples, where close monitoring may be lacking. Capacity building in terms of human resources and infrastructure development in specialized fields would enable sustainable utilization of the available data and resources without dispatching to other regions. All sectors of the country need to play their own role for the success of self-reliance. It is critical for developing countries to have research agendas that address local challenges from diseases and to have future vision to solve current medical problems. National ethics committees in resource limited areas need to establish links with corresponding national and institutional ethics committees in countries where samples are shipped, as a way to safe guard proper use of the shared samples.

## Competing interests

The authors declare that they have no competing interests.

## Authors' contributions

TM, NM, DD and PN conceived the idea for the discussion. TM wrote the original draft manuscript, and incorporated revisions from each of the co-authors. All authors read and approved the final manuscript.

## Declarations

Publication of this supplement has been funded by the College of Health Sciences and the Research Office at the University of Kwazulu-Natal.

This article has been published as part of *BMC Medical Ethics *Volume 14 Supplement 1, 2013: Selected papers from the 3rd Ethics, Human Rights and Medical Law Conference (3rd EHMRL). The full contents of the supplement are available online at http://www.biomedcentral.com/bmcmedethics/supplements/14/S1.
